# *In vivo* DNA methylation editing in zebrafish

**DOI:** 10.1080/15592294.2023.2192326

**Published:** 2023-03-22

**Authors:** Fang Liang, Zijiong Dong, Jianmin Ye, Wei Hu, Ramji Kumar Bhandari, Kangsen Mai, Xuegeng Wang

**Affiliations:** a Institute of Modern Aquaculture Science and Engineering, Guangdong Provincial Key Laboratory for Healthy and Safe Aquaculture, College of Life Sciences, South China Normal University, Guangzhou, P. R. China; b State Key Laboratory of Freshwater Ecology and Biotechnology, Institute of Hydrobiology, Chinese Academy of Sciences, Wuhan, P. R. China; c Department of Biology, University of North Carolina Greensboro, Greensboro, NC, USA

**Keywords:** DNA methylation editing, CRISPR/dCas9, Dnmt7, Tet2, Zebrafish

## Abstract

The CRISPR/dCas9-based epigenome editing technique has driven much attention. Fused with a catalytic domain from Dnmt or Tet protein, the CRISPR/dCas9-DnmtCD or -TetCD systems possess the targeted DNA methylation editing ability and have established a series of *in vitro* and *in vivo* disease models. However, no publication has been reported on zebrafish (*Danio rerio*), an important animal model in biomedicine. The present study demonstrated that CRISPR/dCas9-Dnmt7 and -Tet2 catalytic domain fusions could site-specifically edit genomic DNA methylation *in vivo* in zebrafish and may serve as an efficient toolkit for DNA methylation editing in the zebrafish model.

## Introduction

DNA methylation is a major form of epigenetic modification that regulates various biological processes correlated with the environment, ageing, and disease [1]. In vertebrates, DNA methylation occurs predominantly at the CpG sites and is catalysed by the DNA methyltransferases (DNMT) [2]. Methylated DNA is demethylated in two ways: passive demethylation (also known as replication-based demethylation) and active demethylation, which is a combination of demethylation-related enzymes and mismatch repair-related enzymes, by forming mismatches with oxidation or deamination involving TET family proteins [3]. However, due to a lack of precise epigenome editing tools, a direct causal relationship for investigating a specific gene with a different methylated level has not been fully understood.

The genome targeting technique has dramatically developed with the recent decade’s widespread use of CRISPR/Cas9 systems. The CRISPR/Cas9 complex could target a specific genome site and cut the double-strand DNA chain. The Cas9 protein is a DNA endonuclease that contains two nuclease domains. If both two nuclease domains are inactive, the “dead Cas9 (dCas9)” could bind onto the target sites but do not cut the DNA strands [4]. CRISPR/dCas9 has numerous applications because of its DNA targeting function. The dCas9 can be fused with the catalytic domain of DNA methyltransferases (DNMTs) or ten-eleven translocation (TETs), enabling the CRISPR/dCas9-DNMT or dCas9-TET system with DNA methylation editing functions [5–7].

Taking advantage of the CRISPR/dCas9-based DNA methylation editing system, a series of *in vitro* and *in vivo* disease models have been developed [8,9]. However, the studies applying these epigenome editing techniques *in vivo* are still need to be improved [6,10]. Thus, more animal models are required for *in vivo* editing of the targeted epigenome. Zebrafish (*Danio rerio*) is an excellent vertebrate model for human diseases with its advantages of fast external development, transparency of embryos, and high homology with human genes. To date, little work in epigenome editing has been reported in zebrafish. Here, we demonstrated that CRISPR/dCas9-Dnmt or -Tet catalytic domain fusions are capable of site-specific DNA methylation editing in zebrafish.

## Materials and methods

### Zebrafish husbandry

Wild-type AB-line zebrafish were maintained under standard conditions at 28.5°C, with a 14 h:10 h light:dark cycle. The animal protocols were approved by the Animal Care and Use Committee of the South China Normal University (No. SCNU-SLS-2021-026). All applicable institutional and/or national guidelines for the care and use of animals were followed.

### Plasmid design and construction

**Figure 1.**
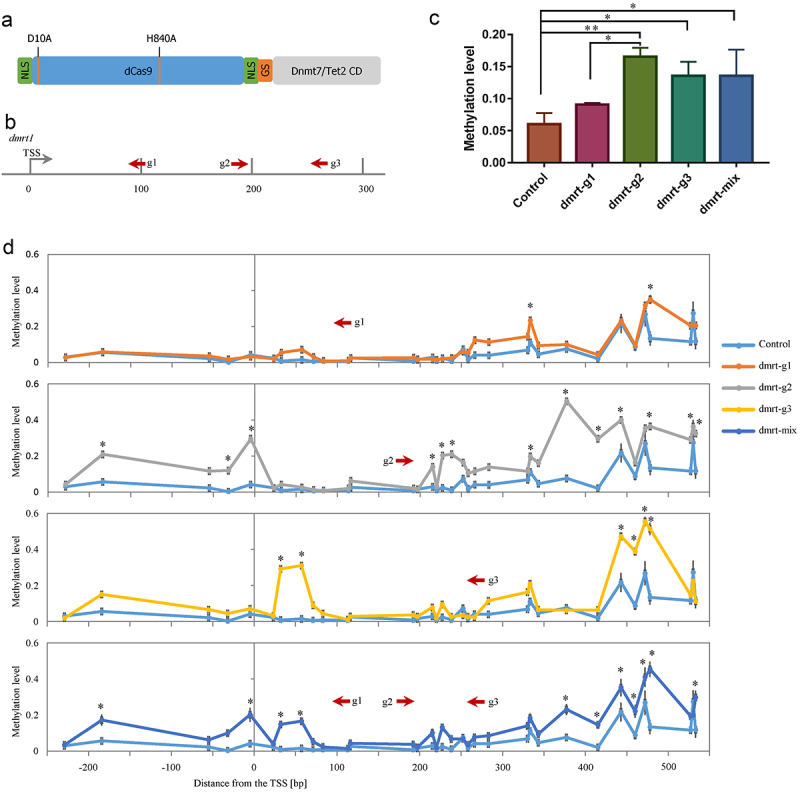
(a) Domain structure of the dCas9–dnmt7/Tet2 catalytic domain fusion protein. The nuclease inactivating mutations D10A and H840A of Cas9 are indicated. Deactivated Cas9 was fused to the catalytic domain of the zebrafish *de novo* DNA methyltransferase 7 (Dnmt7CD) or ten-eleven translocation 2 (Tet2CD) using a short Gly4Ser peptide (GS); (b) Illustration of the locations of the three gRNAs designed to target the upstream regions of the *dmrt1* coding sequence; (c) Average DNA methylation level of target locus within *dmrt1* TSS region (Two-way ANOVA test); (d) Methylation levels of individual CpGs in the TSS region of the *dmrt1* gene. The asterisk indicates statistical significance. (* *p*<0.05).

The dCas9-Dnmt7CD and dCas9-Tet2CD fusion proteins were constructed by adding the Dnmt7 or Tet2 catalytic domain to the C-terminus of the inactive Cas9 (dCas9) via a short Gly4Ser linker (Figure 1a) [5,6,11]. The plasmid pT3TS-nCas9n (Addgene plasmid # 46757) [12], encoding Cas9 protein, was deactivated by introducing the D10A and H840A mutation using the Mut Express II Fast Mutagenesis Kit V2 (Vazyme, C214–02) [4,13]. A Gly4Ser linker (GS) was inserted at the C-terminus of the dCas9 gene. The C-terminal Nuclear Localization Sequence (NLS) and the N-terminal NLS were left intact.

The zebrafish Dnmt7 (also known as Dnmt3ba) catalytic domain (NM_001020476.2) and Tet2 catalytic domain (XM_005159903.4) were amplified from cDNA using the 2 × Phanta Master Mix (Vazyme, P511–02) to enable insertion C-terminally from the Gly4Ser linker.

### mRNA synthesis and microinjection

The dCas9-Dnmt7CD and dCas9-Tet2CD mRNAs were *in vitro* transcribed from *Sfi*I linearized plasmid dCas9-Dnmt7CD and dCas9-Tet2CD templates using the T3 mMESSAGE mMACHINE® Kit (Ambion, AM1348) and were purified using an RNAclean Kit (TIANGEN, DP412). Then, the mRNAs were quantified by NanoDrop 2000 (Thermo Fisher Scientific, USA) and standardized to 1000 ng/μl for stock at −80°C. The promoter and 5’UTR sequence of each target gene was obtained from NCBI and confirmed by the Sanger sequence. gRNAs were selected based on the location of PAM sequences, and the gRNA sequences are shown in Table S1. Previous study reported that three or more interspaced and five concatenated mismatches eliminated Cas9 cleavage function [14]. To avoid off-target effects, the target sites were evaluated with CRISPOR [15], and it was double confirmed that no off-target site was found with mismatches less than 5. All the gRNAs were chemically synthesized and modified by GenScript (Nanjing, China) and dissolved in RNase-free water as 1000 ng/μl stock solution in −80°C. One-cell stage zebrafish embryos were injected with 2 nl of a mixture containing the dCas9-Dnmt7CD or dCas9-Tet2CD mRNA (300 ng/μl) and gRNA (30 ng/μl) with different combinations.

### Sample collection and DNA extraction

After 6, 24, or 48 hours post-fertilization (hpf), injected embryos were collected for extraction of DNA or total RNA. Total DNA/RNA was extracted from three pools of ten randomly collected embryos using Quick-DNA/RNA^TM^ Miniprep Plus Kit (Zymo Research, D7003) according to the user’s instruction. Then, the purified genomic DNA was used for DNA methylation determination.

### Multiplex methylation PCR sequencing

Multiplex methylation PCR (MMP) sequencing was used to determine the methylation status of individual CpG sites located in the selected regions. The MMP sequencing experiments were performed at MethylGene Tech Co., Ltd. Guangzhou. Firstly, multiplex primers were designed using the software MethPrimer [16]. Where there was a CpG site in the primer sequence, we substituted the cytosine with a Y/R base to limit bias. These multiplex primers were ligated with the adaptor sequence for the Illumina platform (F: CCTACACGACGCTCTTCCGATCT; R: TTCAGACGTGTGCTCTTCCGATCT) at the 5’ end *in silicon* before being synthesized by Invitrogen.

Next, using these pre-pooled primers, multiplex methylation PCRs were performed on bisulphite converted DNA (100 ng) by EZ DNA Methylation-Gold™ Kit (Zymo Research) under optimized multiplex PCR conditions. The bisulphite conversion reactions were evaluated by examining the conversion rates on CHH sites. A sample with a conversion efficiency above 99% was used as a template for PCRs. The PCRs were performed with the following components: 2× KAPA2G Fast Multiplex Mix buffer (12.5 μl) (Roche, KK5801), bisulphite converted DNA (8.5 μl), and pooled primers (4 μl). The PCR cycling conditions were used as follows: 99°C 2 min; 99°C 15 sec, 60°C 4 min, 27 cycles; 72°C 10 min; and 4°C hold. The PCR products were purified using Agencourt AMPure XP beads.

Finally, following first PCR clean-up, TruSeq Dual Index Adaptors (Illumina) were ligated to each sample by the second round PCR in 25 μl of reaction mixture with the following components: 2× KAPA2G Fast Multiplex Mix buffer (12.5 μl), purified PCR products (10.5 μl), and Illumina index primer (2 μl). The PCR cycling conditions were used as follows: 98°C 1 min; 98°C 15 sec, 60°C 30 sec, 72°C 30 sec, 18 cycles; 72°C 1 min; and 4°C hold. The libraries were then purified using AMPure XP beads. Following library purification, each library was quantified using the Qubit dsDNA HS Assay Kit (Life Technologies, USA). Qualified libraries were sequenced at a Nova Seq platform with a PE150 strategy.

### Statistical analysis

Results were analysed and plotted using GraphPad and Excel software. Statistical analysis among different groups was performed using Two-way ANOVA and Tukey’s multiple comparisons. The data are presented as mean ± standard error of the mean (SEM), and *p* < 0.05 was considered as significant.

## Results and discussion

To design a flexible system to target DNA methylation in zebrafish, we fused dCas9 to the catalytic domain of the zebrafish *de novo* DNA methyltransferase *dnmt7* or ten-eleven translocation methylcytosine dioxygenase *tet2* using a short Gly4Ser peptide (GS) linker (Figure 1a). The conserved sequences of the catalytic domain were determined by aligning zebrafish Dmnt7 or Tet2 with their mammalian homologous genes. The amino acid sequences of constructed fusion proteins are shown in Supplementary Fig. S1 and S2 for dCas9-Dnmt7CD and dCas9-Tet2CD, respectively.

To test this system, we targeted DNA methylation to the sex-specific genes *dmrt1* and *cyp19a1a*. The preliminary experiment found that the targeted TSS region of *dmrt1* was hypomethylated, while the targeted TSS region of *cyp19a1a* was hypermethylated. We, therefore, tested the dCas9-Dnmt7CD system targeting the *dmrt1* TSS region with three different gRNAs (dmrt-g1, dmrt-g2, and dmrt-g3), which bind in different orientations and at distinct positions (Figure 1b). The dCas9-Dnmt7CD mRNA and gRNAs were introduced to the zebrafish embryos at the single-cell stage. The embryos were allowed to develop for 48 hours before sample collection. We used a multiplex methylation PCR (MMP) sequencing strategy to detect the methylation status of the selected regions (Fig. S3 and Table S2). Compared to the control group, the average methylation levels of all 34 CpG sites within the target region were significantly increased in dmrt-g2 group (0.1656 vs. 0.0600, *p* < 0.01), and dmrt-g3 group (0.1358 vs. 0.0600, *p* < 0.05) (Figure 1c). Though the dmrt-g1 group did not have a significant change in average methylation level compared to the control group (0.0908 vs. 0.0600) (Figure 1c), there were still two individual CpG sites that had significantly increased methylation levels (Figure 1d). Consistent with the average methylation changes, the dmrt-g2 group had 13 significantly increased methylation levels in individual CpG sites, and the dmrt-g3 groups had 6 individual CpG sites that significantly increased methylation levels (Figure 1d).

Subsequently, we tested whether targeting multiple loci with *dmrt1* gRNAs mixture could further increase the methylation levels. The result showed that targeting with all three gRNAs to the *dmrt1* TSS did not result in an increased methylation level (dmrt-mix group, 0.1354) compared to single gRNA targeting experiments (e.g., dmrt-g2 group 0.1656) (Figure 1c). Further analysis showed that there were 11 individual CpG sites significantly increased in methylation levels, which was less than the dmrt-g2 group (Figure 1d).

A comparison of all data showed a high absolute methylation fraction increase right downstream of the PAM site (around+40 bp) (Fig. S4). The second peak of absolute methylation fraction increase was observed right downstream of the PAM site around+200 bp (Fig. S4). On the left upstream of the PAM site, the first peak of absolute methylation fraction increase appeared around −80 bp, and the second peak was observed around −230 bp (Fig. S4). The distance between two nearby peaks is close to the length of mononucleosome fragments (150–175 bp) [17], indicating that the nucleosome structure of the genome may affect the DNA methylation editing. DNA methylation was not introduced directly at the dCas9 binding sites, indicating that binding of dCas9 fusion protein can prevent Dnmt from accessing this locus.

**Figure 2.**
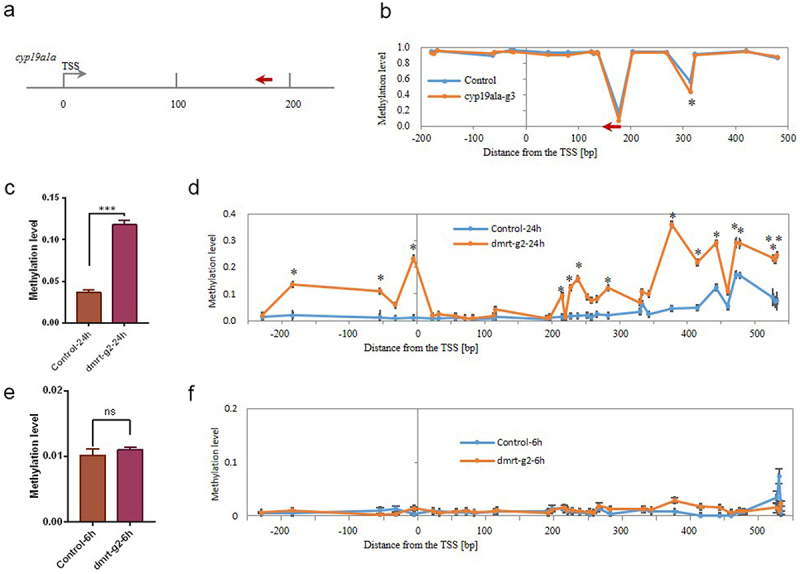
(a) Illustration of the locations of the gRNA designed for targeting the TSS region of the *cyp19a1a* gene; (b) Methylation levels of individual CpGs in the TSS region of the *cyp19a1a* gene; (c) The average DNA methylation level of target locus within the *dmrt1* TSS region at 24 hpf. (d) Methylation levels of individual CpGs in the TSS region of the *dmrt1* gene at 24 hpf. (e) The average DNA methylation level of target locus within *dmrt1*tss region at 6 hpf. (f) Methylation levels of individual CpGs in the TSS region of the *dmrt1* gene at 6 hpf. The asterisk indicates statistical significance. (* *p*<0.05).

Next, we targeted DNA methylation in another sex-specific gene, *cyp19a1a*, with a gRNA targeted within the TSS region (Figure 2a). The dCas9-Tet2CD mRNA and *cyp19a1a* gRNA (cyp19a1a-g3) were co-injected to the zebrafish embryos at the single-cell stage, and the embryos were sampled at 48 hpf. MMP sequencing was used to determine the methylation status of the selected regions (Fig. S5 and Table S2). Compared to the control group, the average methylation levels of all 20 CpG sites within the targeted region did not significantly change in the cyp19a1a-g3 group (0.8612 vs. 0.8808) (Fig. S6A). However, there was still one CpG site that had significantly decreased methylation level (Figure 2b). Nevertheless, the CpG site is not located on the target site of gRNA, indicating that the demethylation event was not introduced by the occupation of the dCas9 fusion protein. Taken together, our results showed that the dCas9-Tet2CD could specifically induce demethylation at the targeted loci.

To investigate the time when these two systems worked, we collected samples at 6 and 24 hpf from dmrt-g2 and cyp19a1a-g3 groups and examined the methylation levels with the MMP sequencing. Results showed that co-injection of dCas9-Dnmt7CD mRNA and dmrt-g2 gRNA could increase DNA methylation by 24 hpf. The average methylation levels in the *dmrt1* TSS region significantly increased in the dmrt-g2 group, compared to the control group at 24 hpf (0.1184 vs. 0.037, *p* < 0.001) (Figure 2c). Consistent with the average methylation changes, the dmrt-g2 group had 15 significantly increased methylation levels in individual CpG sites at 24 hpf (Figure 2d). However, the methylation levels in the *dmrt1* TSS region did not have significant changes at 6 hpf (Figure 2e,f). Results showed that co-injection of dCas9-Tet2CD mRNA and cyp19a1a-g3 did not change the average methylation level within the *cyp19a1a* TSS region at 24 and 6 hpf (Figure S6B and S6C). However, there was an individual CpG site that had significantly decreased the methylation level at 24 hpf (Fig. S7A), but not at 6 hpf (Fig. S7B). Furthermore, the CpG site was the same one identified at 48 dpf, indicating that the demethylation state had been established at 24 hpf and persisted until 48 hpf. These results suggest that the CRISPR/dCas9-mediated DNA methylation editing system can methylate or demethylate CpG sites as early as 24 hpf.

The present work utilized for the first time CRISPR/dCas9-DnmtCD or -TetCD fusions to site-specifically edit the DNA methylation in zebrafish. As a promising alternative animal model, these tools hold great potential to broaden the usage of zebrafish in understanding epigenetic regulation of development, health, and disease.

In mammals, there are two types of DNA methyltransferases involved in DNA methylation regulation: DNMT1 and DNMT3 family. All of them have been used in site-specific DNA methylation editing [8]. In zebrafish, *dnmt3*, *dnmt4*, *dnmt5,* and *dnmt7* (also known as *dnmt3ba*) are homologous genes of mammalian *Dnmt3b*, whereas *dnmt6* and *dnmt8* are homologous to mammalian *Dnmt3a*. All six genes are expressed by the blastula stage in zebrafish [18]. Among them, zebrafish *dnmt7* was demonstrated to be maternally expressed [19] and was used in the present study. Although the present study achieved a high DNA methylation editing efficiency with Dnmt7 catalytic domain, the other Dnmts should also be tested to find the most suitable DNA methyltransferase. We did not observe a high efficiency in CRISPR/dCas9-Tet2CD-induced DNA demethylation in the present study. In zebrafish, unlike the mice and humans, the zygotic genome does not undergo a demethylation process [20], and all *tet1*, *tet2,* and *tet3* are barely expressed before 6 hpf [21,22]. Among them, *tet2* looks the most expressed during embryogenesis and universal in tissues and is used in the present study [21]. Due to the absence of Tet’s expression at early embryogenesis, it probably lacks a suitable environment required for Tet2 function before 6 hpf. Also, with the division of embryonic cells, the dCas9-Tet2CD mRNA and gRNA were diluted into daughter cells and may have lost their function gradually when the environment is ready for Tet2 to function properly. It is critical to find an alternative for Tet2, which expresses and functions at the cleavage stage. For instance, *unga* has been found to be involved in post-fertilization genomic DNA demethylation in zebrafish [23], which may serve as an alternative functional catalytic domain in the fusion protein.

In the present study, we observed the first methylation peak at around+40 bp downstream of the PAM sequence, which is longer than previous studies [7,24]. Vojta *et al*. reported a peak centred at 27 bp downstream from the PAM sequence when using a catalytic domain from human Dnmt3a [7]. Stepper *et al*. reported the highest peak appeared at 25 bp distance from PAM sequence when using a chimeric catalytic domain from murine Dnmt3a and Dnmt3L [24]. On the other hand, we observed a peak around −80 bp from the PAM sequence, while Stepper *et al*. reported the peak centred around −40 bp from the PAM sequence [24]. We could hypothesize that the differences in the peak location are probably because of the length of the linker and catalytic domain and their higher structures. It is critical to understand the peak locations of both sides from the PAM sequence to obtain the best DNA methylation editing efficiency on the target site or region. Another crucial issue in the epigenome editing is the higher structure of genome DNA. In the present study and previous study [24], multiple peaks with distance close to the length of mononucleosome were observed, suggesting that the mononucleosome structure could affect the function of the CRISPR/dCas9-based epigenome editing systems. These results raise another crucial question of how to restrict the epigenome editing systems function within a designed region and require more research to improve the epigenome editing approaches.

Increasing evidence has proven that *dmrt1* and *cyp19a1a* play essential roles in the sex determination in aquaculture animals [25]. The development of epigenome editing tools holds potential usage to obtain unisexual aquaculture species. In recent years, the potential application of epigenetic mechanisms in aquaculture has driven researchers’ attention and undergone extensive discussion [26]. However, the topics majorly focus on the inheritable epigenetic signatures induced by environmental conditions, such as temperature, nutrition, hypoxia, environmental chemicals, and metals. The strategy, i.e., “forward epigenetics,” relies on the “environment−gene” interaction and is from phenotype to epigenotype. In the present study, we developed a promising tool that could change the epigenotype directly. The tool enables us to investigate the application of epigenetic mechanisms in a different strategy. This strategy, i.e., “reverse epigenetics,” is from epigenotype to phenotype and holds great potential to broaden our toolkit in aquaculture breeding.

## Conclusions

Here, we report an effective *in vivo* site-specific DNA methylation editing system in zebrafish, which may serve as an efficient toolkit to study epigenetic regulation of development, health, and disease and enhance our understanding of the causal relationship between DNA methylation and the expression of a specific gene.

## Supplementary Material

Supplemental Material

## Data Availability

The sequence data generated in this study is available at the Genome Sequence Archive (GSA), under accession number CRA006715, which is accessible at https://ngdc.cncb.ac.cn/gsa.
