# Supplementary material for: In vivo DNA methylation editing in zebrafish
Source: Epigenetics. 2023 Mar 22;18(1):2192326. doi: 10.1080/15592294.2023.2192326 (PMC10038036; doi:10.1080/15592294.2023.2192326)

**Supplementary Information**

# *In vivo* DNA methylation editing in zebrafish

Fang Liang^a^, Zijiong Dong^a^, Jianmin Ye^a^, Wei Hu^b^, Ramji Kumar Bhandari^c^, Kangsen Mai^a^, Xuegeng Wang^a *^

^a^ Institute of Modern Aquaculture Science and Engineering, Guangdong Provincial Key Laboratory for Healthy and Safe Aquaculture, College of Life Sciences, South China Normal University, Guangzhou 510631, P. R. China

^b^ State Key Laboratory of Freshwater Ecology and Biotechnology, Institute of Hydrobiology, Chinese Academy of Sciences, Wuhan 430072, P. R. China

^c^ Department of Biology, University of North Carolina Greensboro, Greensboro, NC 27412, USA

^*^ Corresponding Author: Xuegeng Wang (Email: wangxuegeng@scnu.edu.cn)

# Supplementary Tables

**Table S1**. sgRNAs

| **Name** | **Target** | **Target site sequence including PAM (red)** |
| --- | --- | --- |
| dmrt-g1 | dmrt1, TSS | GCGCTTATAAATAGGGTCTGAGG |
| dmrt-g2 | dmrt1, TSS | GTGAAGAAGAGCAGACTAACGGG |
| dmrt-g3 | dmrt1, TSS | TCAGCGGTGACACGAAGCCGTGG |
| cyp19ala-g3 | cyp19ala, TSS | CAGTGCCGTTATGAGCCCTTTGG |

**Table S2**. Oligonucleotides

| **Name** | **Sequence (5'-3')** | **Purpose** |
| --- | --- | --- |
| dmrt1-MMP-F1 | GAATATGATAAATGAAGGTGTATTAAA | MMP amplificon |
| dmrt1-MMP-R1 | ATCAAAATAATTCAAAAATCAAAATAAAAA | MMP amplificon |
| dmrt1-MMP-F2 | GAGTTTTATGTAGTTGTTGGGATATAAA | MMP amplificon |
| dmrt1-MMP-R2 | AACAAACAAAATAACAACAACAATA | MMP amplificon |
| dmrt1-MMP-F3 | AATTATATATTTTAGGTTAGTTGGATTTG | MMP amplificon |
| dmrt1-MMP-R3 | ATATAATTAAATTCATTTCTTAAAACTT | MMP amplificon |
| dmrt1-MMP-F4 | TATTGAAAGGGTTTAGGATAATG | MMP amplificon |
| dmrt1-MMP-R4 | TCTAAAACAAATCCAACTAACCTAAAATA | MMP amplificon |
| cyp19a1a-MMP-F1 | GAATGTTGTTTTATGTGTTTAAAAAAG | MMP amplificon |
| cyp19a1a-MMP-R1 | ATAAAAACTACAACCCATTTATTAT | MMP amplificon |
| cyp19a1a-MMP-F2 | TTGGGAGTTTTAGTGTGAAGGAG | MMP amplificon |
| cyp19a1a-MMP-R2 | ACCAATCTACTCTACTCTAAATAAAAATTA | MMP amplificon |
| cyp19a1a-MMP-F3 | TTATTATTAGTTGTTAGGATGA | MMP amplificon |
| cyp19a1a-MMP-R3 | CTACATTTCTAACACTAACA | MMP amplificon |
| cyp19a1a-MMP-F4 | GGTTGTTTAGGTAATTTAGATATTTT | MMP amplificon |
| cyp19a1a-MMP-R4 | TTTATCAAAACAATTATAACAAACAATTA | MMP amplificon |
| cyp19a1a-MMP-F5 | GTAGGTTTATTATTTTTTAATATGGGTGA | MMP amplificon |
| cyp19a1a-MMP-R5 | AAAAACCTATACATTCCAAAACT | MMP amplificon |
| cyp19a1a-MMP-F6 | TGTAATTGGAGAGATTGTTAGTGTTAGAA | MMP amplificon |
| cyp19a1a-MMP-R6 | AATACAAAATAACCTCTCATAACCTT | MMP amplificon |
| D10A-F | GCCTGGcTATTGGAACTAACTCCGTGGGTTG | Cas9 D10A mutation |
| D10A-R | AGTTCCAATAgCCAGGCCGATGCTATACTTCTT | Cas9 D10A mutation |
| H840A-F | TACGACGTGGATgcTATCGTCCCCCAGAGTTTCATC | Cas9 H840A mutation |
| H840A-R | GATAgcATCCACGTCGTAATCAGACAGCC | Cas9 H840A mutation |
| MCS-5p | aggatccgagctcgctagcctcgagTGACCGCGGATCTGGTTACC | Introducing GS linker and multiple clone site (MCS) |
| MCS-3p | tagcgagctcggatcctccaccgccCACCTTTCTCTTCTTCTTAGGAGATCT | Introducing GS linker and multiple clone site (MCS) |

# Supplementary Figures

**Figure S1**. Amino acid sequences of constructed fusion proteins dCas9-Dnmt7CD.

MASPPKKKRKVGSMDKKYSIGLAIGTNSVGWAVITDDYKVPSKKFKVLGNTDRHSIKKNLIGALLFGSGETAEATRLKRTARRRYTRRKNRICYLQEIFSNEMAKVDDSFFHRLEESFLVEEDKKHERHPIFGNIVDEVAYHEKYPTIYHLRKKLADSTDKADLRLIYLALAHMIKFRGHFLIEGDLNPDNSDVDKLFIQLVQIYNQLFEENPINASRVDAKAILSARLSKSRRLENLIAQLPGEKRNGLFGNLIALSLGLTPNFKSNFDLAEDAKLQLSKDTYDDDLDNLLAQIGDQYADLFLAAKNLSDAILLSDILRVNSEITKAPLSASMIKRYDEHHQDLTLLKALVRQQLPEKYKEIFFDQSKNGYAGYIDGGASQEEFYKFIKPILEKMDGTEELLVKLNREDLLRKQRTFDNGSIPHQIHLGELHAILRRQEDFYPFLKDNREKIEKILTFRIPYYVGPLARGNSRFAWMTRKSEETITPWNFEEVVDKGASAQSFIERMTNFDKNLPNEKVLPKHSLLYEYFTVYNELTKVKYVTEGMRKPAFLSGEQKKAIVDLLFKTNRKVTVKQLKEDYFKKIECFDSVEISGVEDRFNASLGAYHDLLKIIKDKDFLDNEENEDILEDIVLTLTLFEDRGMIEERLKTYAHLFDDKVMKQLKRRRYTGWGRLSRKLINGIRDKQSGKTILDFLKSDGFANRNFMQLIHDDSLTFKEDIQKAQVSGQGHSLHEQIANLAGSPAIKKGILQTVKIVDELVKVMGHKPENIVIEMARENQTTQKGQKNSRERMKRIEEGIKELGSQILKEHPVENTQLQNEKLYLYYLQNGRDMYVDQELDINRLSDYDVDAIVPQSFIKDDSIDNKVLTRSDKNRGKSDNVPSEEVVKKMKNYWRQLLNAKLITQRKFDNLTKAERGGLSELDKAGFIKRQLVETRQITKHVAQILDSRMNTKYDENDKLIREVKVITLKSKLVSDFRKDFQFYKVREINNYHHAHDAYLNAVVGTALIKKYPKLESEFVYGDYKVYDVRKMIAKSEQEIGKATAKYFFYSNIMNFFKTEITLANGEIRKRPLIETNGETGEIVWDKGRDFATVRKVLSMPQVNIVKKTEVQTGGFSKESILPKRNSDKLIARKKDWDPKKYGGFDSPTVAYSVLVVAKVEKGKSKKLKSVKELLGITIMERSSFEKNPIDFLEAKGYKEVKKDLIIKLPKYSLFELENGRKRMLASAGELQKGNELALPSKYVNFLYLASHYEKLKGSPEDNEQKQLFVEQHKHYLDEIIEQISEFSKRVILADANLDKVLSAYNKHRDKPIREQAENIIHLFTLTNLGAPAAFKYFDTTIDRKRYTSTKEVLDATLIHQSITGLYETRIDLSQLGGDSPVRSPKKKRKVGGGGSELSIPAHKRRPIRVLSLFDGIATGYLVLKDLGFKLERYIASEICGDSIAVGMVKHEGKIEYVKDVRTITRKHLAEWGPFDLLIGGSPCNDLSMVNPARKGLFEGTGRLFFEYYRMLTMMRPKEDDDRPFFWLFENVVAMSAHDKADICRFLECNPVMIDAVKVSPAHRARYFWGNLPGMNRPVATSLTDNVDLQDCLESGRTAMFSKVRTITTKSNSIKQGKTGPLPVTMNGKEDYLWCTEMEKIFGFPKHYTDVNNMGRGQRQKVLGRSWSVPVIRHLFAPLKDYFACEASLE.

Legend: NLS; dCas9; Gly4Ser linker; Dnmt7 catalytic domain

**Figure S2**. Amino acid sequences of constructed fusion proteins dCas9-Tet2CD

MASPPKKKRKVGSMDKKYSIGLAIGTNSVGWAVITDDYKVPSKKFKVLGNTDRHSIKKNLIGALLFGSGETAEATRLKRTARRRYTRRKNRICYLQEIFSNEMAKVDDSFFHRLEESFLVEEDKKHERHPIFGNIVDEVAYHEKYPTIYHLRKKLADSTDKADLRLIYLALAHMIKFRGHFLIEGDLNPDNSDVDKLFIQLVQIYNQLFEENPINASRVDAKAILSARLSKSRRLENLIAQLPGEKRNGLFGNLIALSLGLTPNFKSNFDLAEDAKLQLSKDTYDDDLDNLLAQIGDQYADLFLAAKNLSDAILLSDILRVNSEITKAPLSASMIKRYDEHHQDLTLLKALVRQQLPEKYKEIFFDQSKNGYAGYIDGGASQEEFYKFIKPILEKMDGTEELLVKLNREDLLRKQRTFDNGSIPHQIHLGELHAILRRQEDFYPFLKDNREKIEKILTFRIPYYVGPLARGNSRFAWMTRKSEETITPWNFEEVVDKGASAQSFIERMTNFDKNLPNEKVLPKHSLLYEYFTVYNELTKVKYVTEGMRKPAFLSGEQKKAIVDLLFKTNRKVTVKQLKEDYFKKIECFDSVEISGVEDRFNASLGAYHDLLKIIKDKDFLDNEENEDILEDIVLTLTLFEDRGMIEERLKTYAHLFDDKVMKQLKRRRYTGWGRLSRKLINGIRDKQSGKTILDFLKSDGFANRNFMQLIHDDSLTFKEDIQKAQVSGQGHSLHEQIANLAGSPAIKKGILQTVKIVDELVKVMGHKPENIVIEMARENQTTQKGQKNSRERMKRIEEGIKELGSQILKEHPVENTQLQNEKLYLYYLQNGRDMYVDQELDINRLSDYDVDAIVPQSFIKDDSIDNKVLTRSDKNRGKSDNVPSEEVVKKMKNYWRQLLNAKLITQRKFDNLTKAERGGLSELDKAGFIKRQLVETRQITKHVAQILDSRMNTKYDENDKLIREVKVITLKSKLVSDFRKDFQFYKVREINNYHHAHDAYLNAVVGTALIKKYPKLESEFVYGDYKVYDVRKMIAKSEQEIGKATAKYFFYSNIMNFFKTEITLANGEIRKRPLIETNGETGEIVWDKGRDFATVRKVLSMPQVNIVKKTEVQTGGFSKESILPKRNSDKLIARKKDWDPKKYGGFDSPTVAYSVLVVAKVEKGKSKKLKSVKELLGITIMERSSFEKNPIDFLEAKGYKEVKKDLIIKLPKYSLFELENGRKRMLASAGELQKGNELALPSKYVNFLYLASHYEKLKGSPEDNEQKQLFVEQHKHYLDEIIEQISEFSKRVILADANLDKVLSAYNKHRDKPIREQAENIIHLFTLTNLGAPAAFKYFDTTIDRKRYTSTKEVLDATLIHQSITGLYETRIDLSQLGGDSPVRSPKKKRKVGGGGSEIPSCHCVEQISERDEGPYYTHLGSASNVAGIRKIMEERSGMTGSAIRIEKVLYTGKEGKSGLGCPIAKWVIRRANEDEKILVLVRERAGHSCETSCVVVVILIWEGIPTSLADRLYMELSDTLTKHGALTNRRCALNEERTCACQGLEADACGASFSFGCSWSMYYNGCKFARSKVPRKFKLLADDPKEEEKIEQNLQGLATYIAPVYKKMAPDAYSNQVEHENRAPDCRLGLKEGRPFSGVTACLDFCAHAHRDLHNMQGGSTVVCTLTREDNREIGKIPEDEQLHVLPLYKPSSTDEFGSAEAQLEKTKTGAIQVLSSFRRQVRMLPEPAKSCRQRKLDAKRANKPNNNTPNSKTDNTQQAKQKQTAYENPTVTGRGNMRTNLDSGHLPQAHAGHQPQQQFPQQTHPNPSYASPPFTRFPNASKPSTPHPQTPSVDPYASPLHAPNSYINASNAPSPYSRSLAPSPLYNGYQCNGGIPMDNYHPYHSSNLKHPDMFHPQRNPLYSEQQYNAPQHYGVNYPPHYGEAMATNGYGNCNMRPGIHSMGHYPGFDSNMSTNAFARPPSAHLHLDYAAAGNAYPKPHISQNPHMFSPNLNTLSMQSHKDLGISMHEANGISQGFPPLGKECFNFNQPSSLKLPNENAHNPAVNPTQLPQVSEKKEEDVWSDSEHNFLDPEIGGVAVAPSHGSIIIECAKRELHATTPVKKPDRNHPTRISLVFYQHKNLNEAKHGLSLWEAKMAEKAREKEEDAEKHGAENTSSKSGGKKAKREHSEHSEPSEPPYKQFLLMLTERSMSCTTNTYVSTSPYAFTKVTGPYNNFM.

Legend: NLS; dCas9; Gly4Ser linker; Tet2 catalytic domain

**Figure S3**. Genomic sequence of the *dmrt1* locus with gRNA sequences labeled in black and CpGs in box. PAM for each gRNA is highlighted in red. The multiplex methylation PCR amplicon is highlighted by underline. Gene structure annotation was downloaded from the UCSC Genome Browser.

MMP amplicon; **gRNA**; PAM; CpG site

**Figure S4**. Summary profile of the dCas9-Dnmt7CD activity is shown as an absolute methylation fraction increase relative to the distance of the PAM sequence.

**Figure S5**. Genomic sequence of the *cyp19a1a* locus with gRNA sequences labeled in black and CpGs in box. PAM for gRNA is highlighted in red. The multiplex methylation PCR amplicon is highlighted by underline. Gene structure annotation was downloaded from the UCSC Genome Browser.

MMP amplicon; **gRNA**; PAM; CpG site

**Figure S6**. (A) The average DNA methylation level of target locus within *cyp19a1a* TSS region at 48 hpf. (B) The average DNA methylation level of target locus within *cyp19a1a* TSS region at 24 hpf. (C) Average DNA methylation level of target locus within *cyp19a1a* TSS region at 6 hpf.


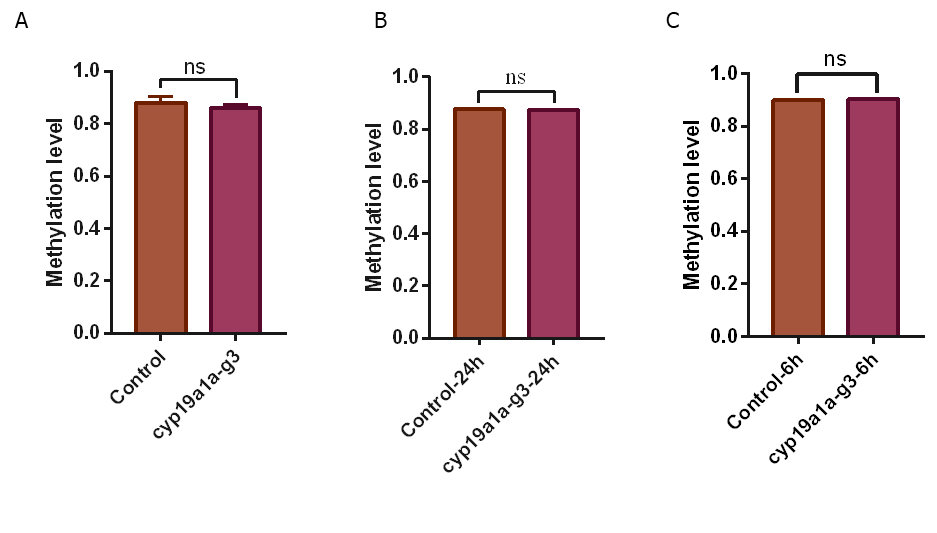


**Figure S7**. Methylation levels of individual CpGs in the TSS region of the *cyp19a1a* gene at 24 hpf. (B) Methylation levels of individual CpGs in the TSS region of the *cyp19a1a* gene at 6 hpf. Asterisk indicates statistical significance (* *p* < 0.05).


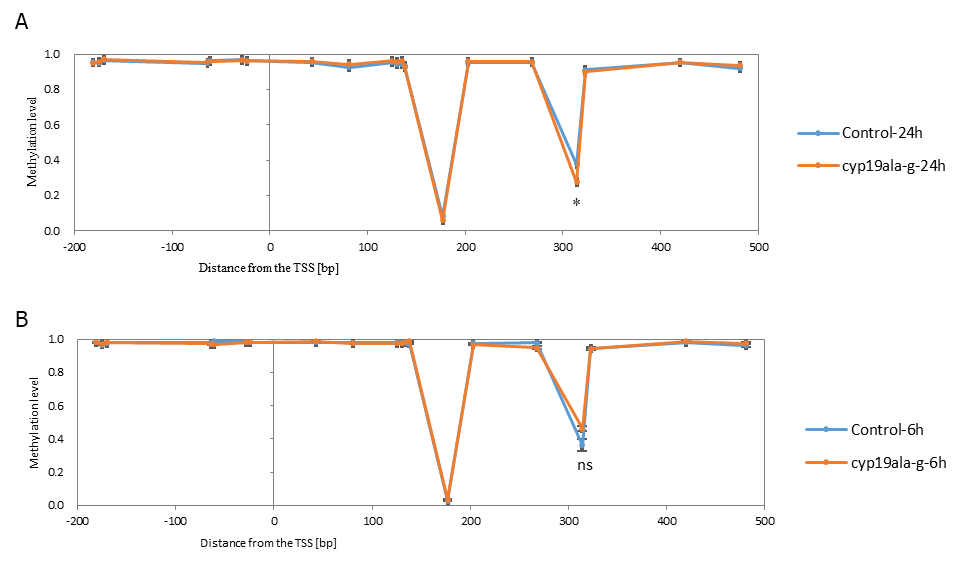

Supplement: Supplemental Material [file KEPI_A_2192326_SM0413.docx]
